# Novel advances in juvenile idiopathic arthritis associated uveitis

**DOI:** 10.1186/s13075-026-03758-1

**Published:** 2026-02-27

**Authors:** Anamika Patel, Nisha R. Acharya, Ameenat Lola Solebo

**Affiliations:** 1 Great Ormond Street Hospital NHS Trust, London, UK; 2 Moorfields Eye Hospital NHS Trust, London, UK; 3 Francis I Proctor Foundation, University of California, San Francisco, CA USA; 4 Department of Ophthalmology, University of California, San Francisco, CA USA; 5 Population, Policy and Practice Department, UCL GOS Institute of Child Health, Room 5.02, 30 Guilford Street, London, WC1N 1EH UK; 6 Great Ormond Street NIHR Biomedical Research Centre, London, UK

**Keywords:** Juvenile idiopathic arthritis, Uveitis, Biomarkers, Biologic therapy, Diagnosis

## Abstract

**Introduction:**

Juvenile idiopathic arthritis–associated uveitis (JIA-U) is an important cause of childhood and adult visual impairment. Advances in surveillance approaches and treatment have improved outcomes, yet delayed detection and treatment-refractory disease remain substantial challenges. This review summarises recent developments across disease stratification, imaging, pharmacologic management, and psychosocial support, following the natural history of disease from inception to adulthood.

**Main body:**

Risk-stratified screening based on antinuclear antibody status, JIA subtype, and age at arthritis onset is now standard, but many cases present outside screening windows. Novel biomarkers, including S100A8/A9, S100A12, and genetic risk alleles, offer promise for earlier identification. Ocular imaging modalities such as anterior segment optical coherence tomography and optical coherence tomography angiography enable objective, child-friendly detection of inflammation and subclinical vascular changes, potentially extending specialist-level assessment to community settings.

Early initiation of immunomodulatory therapy in children with JIA is now prevalent care, reducing uveitis incidence and improving outcomes in childhood, although the consequence may be new challenges later in the lifecourse. Anti-tumour necrosis factor therapy has changed the management of JIA-U for the better, although precision strategies, such as anti-drug antibody monitoring, dose individualisation, and tapering approaches are needed to further optimise care. For refractory disease, biologics and other emerging therapies are under investigation, but new therapeutic targets are needed.

Beyond ocular health, JIA-U imposes a heavy psychosocial burden on children and families, exacerbated by treatment side-effects and frequent medical visits. Validated tools, such as the EYE-Q questionnaire, and co-developed educational and self-management resources will be key to addressing mental health and quality-of-life concerns.

**Conclusion:**

JIA-U remains a lifelong, vision-threatening condition despite advances in screening, diagnostics, and treatment. Integration of biomarker-based risk stratification, advanced imaging, and precision pharmacology holds promise for earlier detection and personalised care. Addressing psychosocial impacts through family-centred, multidisciplinary frameworks is essential. Future priorities include validating predictive biomarkers, refining tapering protocols, and ensuring equitable access to novel diagnostics and therapeutics to optimise lifelong outcomes.

## Background

**Fig. 1:**
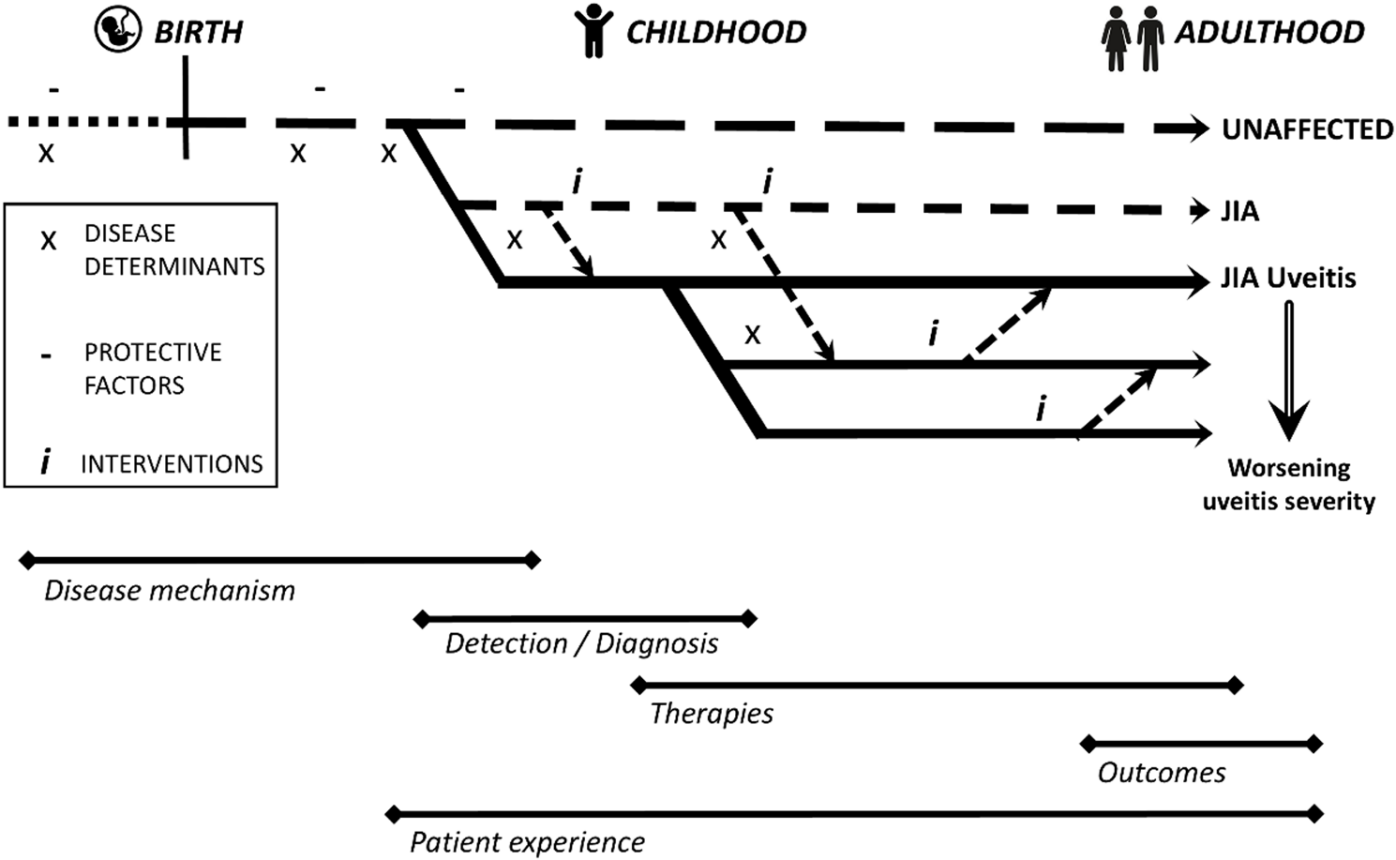
A framework for disease trajectory in children with juvenile idiopathic associated uveitis, and the areas of focus in this review

Juvenile idiopathic arthritis-associated uveitis, or JIA-U, is a potentially disabling disorder. Occurring in approximately 20% of children with JIA [1], uveitis is one of the most common extra-articular manifestations of disease. The often insidious onset of JIA-U allows damage to progress unnoticed prior to diagnosis [2–4]. The occurrence of that damage confers great burden: childhood onset uveitis remains an important cause of visual impairment [5–7]. The threat to vision at a young age profoundly impacts later life development. Patients who manage to escape visual loss during childhood remain at risk of further sight loss as adults [8, 9], with a disproportionately large prevalence of childhood onset disease amongst those with uveitis related disability [7]. In this review, we will describe the advances which have deepened our understanding of JIA-U, by following the journey of a child with JIA-U from diagnosis to adulthood (Fig. 1).

Over the past two decades, clinical strategies in JIA-U have shifted toward interventions for targeted surveillance in order to ensure earlier detection. Risk-stratified screening protocols, based on ANA status, JIA subtype, and age at arthritis onset, have been introduced, supported by national and international consensus [10–13]. Many children still present outside screening windows, even in higher income settings, resulting in avoidable visual disability [14]. We will address the attempts to improve the pathways to prompt detection for affected children, and we will review the emerging predictive tools, including serologic markers and genetic profiling, that may enable earlier identification of those at risk [15]. 

Early initiation of methotrexate and timely escalation to anti-TNF agents like adalimumab have become standard [11, 16, 17]. Recent cohort data suggests that this reduces the occurrence of uveitis, or improves ocular and visual outcomes in incident cases [16]. Yet, challenges remain. Treatment refractory disease is a concern. While many respond to adalimumab, a currently unpredictable proportion of patients fail to achieve sustained disease control or remission [15, 18–21]. Strategies like drug level monitoring and early dose adjustments show promise but are not universally accessible [22]. For non-responders, JAK inhibitors and biologics like abatacept and tocilizumab are being explored [23, 24]. This review will discuss the current evidence base around JIA-U therapies.

Uveitis often persists into adulthood. We review the available data on long-term visual and systemic outcomes, and the implicated need for coordinated transitional care. Affected children, their families and their clinical teams must battle the tension of the spectre of ‘silent’, or largely asymptomatic ocular inflammation which can result in blinding complications, frequent hospital visits with travel to distant specialist centers, and the all-too often negative experience of taking the multiple immunosuppression agents needed to prevent irreversible vision loss [25, 26]. In this review, we turn to the psychosocial dimensions, and the value of co-developed or patient-led resources. Together, these perspectives highlight the need for multidisciplinary, personalised care rooted in evidence, but equally responsive to the evolving realities of growing up with the lifelong spectre of a vision-threatening disorder.

### Identifying children at risk of JIA uveitis: the future role of serological markers, ocular fluid and DNA

Serological markers of ocular inflammation are potentially useful tools for population stratification, prompt disease detection and for an expansion of our understanding of disease pathogenesis. Anti-nuclear antibody positivity is a robust marker of future uveitis risk in JIA [1, 27], although there are confounding relationships with age and sex, and ANA negativity does not remove the risk of uveitis. S100A8/A9 (calprotectin) and S100A12 are myeloid-derived damage-associated molecular pattern (DAMP) initially recognized as indicators of systemic disease activity in JIA subtypes [28], but which are now well-documented in ocular inflammation, even when the arthritis is in long term remission [29]. These proteins, however, are not disease-specific [28, 30, 31]. Clinical utility is further constrained by inter-individual variability and systemic confounders such as the use of DMARDs (disease modifying anti-rheumatic drugs) [28, 29, 32]. 

Ocular fluid sampling offers a direct view into intraocular immune mechanisms. Whilst it is unlikely that invasive intraocular sampling of aqueous humour (AqH) will be adopted for routine care, the concordance and discordance noted between serological and AqH samples support or oppose postulations of biomarker utility for population stratification. For example, there is an absence of consistent correlation of systemic and intraocular S100 levels, possibly due to compartmentalized immune responses, treatment effects, or blood-ocular barrier integrity [33]. Tear fluid sampling, by contrast, is non-invasive and thus particularly suitable for children. Proteomic analysis of paired samples has identified multiple overlaps across the proteins identified in aqueous and tears [34, 35]. Markedly higher levels of S100A12 have been found in the tears of children with active JIA-U compared to those with inactive disease [33, 35, 36], suggesting a possible future role in detecting those at risk. Elevated tear IL-8 and soluble ICAM-1 tear levels are other putative markers of disease activity in JIA-U, although they appear less able to differentiate between those with or without uveitis [35], limiting their usefulness for disease detection. Larger scale longitudinal tear sample proteomic studies in children diagnosed with JIA prior to the development of uveitis, and studies with serial sampling across the natural history of JIA-U, should enable interrogation of the power of these markers in predicting outcomes.

Novel understanding of the genetic risk profile for uveitis in JIA brings an additional tool for potential patient stratification, focusing clinical attention on those most at risk. The addition of three independent genetic risk factors for JIA-U (mapping to *HLA-DRB1*, *HLA‐DPB1*, and *HLA‐A)* to a predictive risk algorithm which also included ANA status, age at onset and ILAR category, improved the algorithm performance over use of the clinical variables alone [37]. However, even with this improved performance, the area under the receiver operator characteristic curve (AUC) for the model was below the 0.80 threshold typically indicative of clinical utility [38]. Whilst JIA and JIA-U are thought to be complex (or multifactorial) genetic disorders, there is still only modest support for the role of non-HLA genes in disease pathogenesis. Genome-wide association studies (GWAS) have identified associations between JIA-U risk and loci within, for example, the tumor necrosis factor receptor-associated factor 1/complement component 5 (TRAF1-C5), V-set domain containing T-cell activation inhibitor-1 (VTCN1), interferon regulatory factor 5 (IRF5), mediterranean fever (MEFV), and the Protein Tyrosine Phosphatase Non-Receptor Type 2 (PTPN2) genes or genetic regions [39–41]. 

### The role of ocular imaging in disease detection

**Fig. 2:**
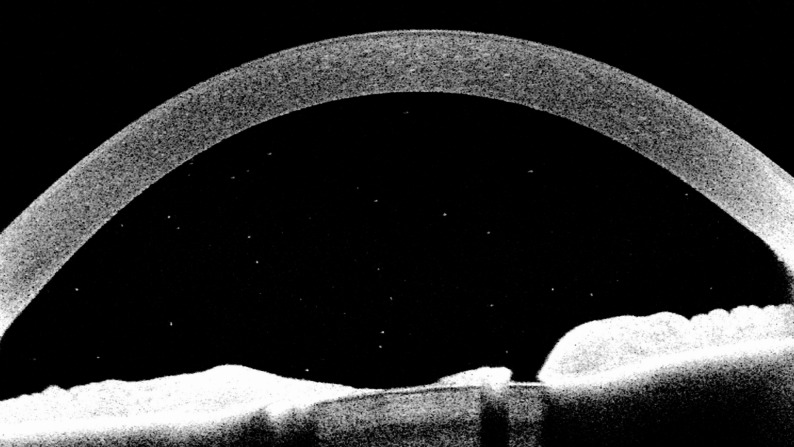
Cross sectional (axial) AS-OCT image of the ocular anterior chamber of a child with active JIA-U. Inflammatory cells appear as hyper-reflective particles between the hyperreflective dome of the cornea anteriorly and iris/lens diaphragm posteriorly

Advanced imaging modalities are emerging as vital tools for detecting and monitoring intraocular inflammation in JIA-U. Anterior segment optical coherence tomography (AS-OCT) provides high-resolution cross-sectional images of the anterior chamber [42], allowing objective visualization of intraocular inflammatory cells (Fig. 2). This addresses the inherent subjectivity and examiner dependency of the current assessment method [43], slit-lamp examination, and provides expert level assessment in the increasingly common scenario of slit lamp examination being impossible due to the absence of a trained ophthalmic specialist [44–46]. This expert level assessment is particularly needed in light of the worsening global shortfall in paediatric ophthalmologist and uveitis specialists [46–48]. 

Cross-sectional studies have confirmed that AS-OCT is both sensitive and specific for detecting anterior uveitis [49–53]. In a cohort of 150 children with JIA, AS-OCT detection of clinical activity had a sensitivity of 94%, specificity of 96%, and a negative predictive value of 0.99 [49]. Recent innovations, such as particle size-based analysis, enhance specificity: for example, AS-OCT now appears to be able to differentiate inflammatory cells and pigment debris [54], and may be able to characterise inflammatory cell populations [51, 55], with different reflectivity signals generated by lymphocytes, neutrophils and monocytes [56]. The paediatric uveitis population also benefits from normative data gained through large studies of healthy eyes [57]. As a non-invasive, non-contact, non-irradiating and widely accessible digital imaging technology, AS-OCT is highly acceptable to, and welcomed, by children and families as a future disease monitoring tool, able to make ‘the invisible, visible’ to those affected by this insidious disorder [50, 51]. A randomized trial is currently underway to assess the real-world applicability of AS-OCT as a screening tool for JIA-U [50, 51, 58]. 

Complementing AS-OCT, optical coherence tomography angiography (OCTA) provides non-invasive, dye-free imaging of the retinal and choroidal vasculature. OCTA has identified reduced retinal vessel density and choriocapillaris flow in JIA patients with and without uveitis, suggesting microvascular changes which precede clinical inflammation or reflect systemic vasculopathy. Together, AS-OCT and OCTA offer a comprehensive imaging framework: AS-OCT quantifies anterior inflammation, while OCTA reveals deeper vascular alterations. Incorporating these modalities into clinical care may improve early detection, reduce diagnostic variability, and support individualized monitoring. Digital technologies such as OCT and OCTA imaging are also particularly well suited for future use in multimodal machine learning models which might effectively predict therapeutic response and outcomes. Barriers such as equipment costs, training demands, and healthcare infrastructure will need to be addressed to ensure equitable implementation across diverse settings.

### Optimising Anti-TNF therapy in JIA- uveitis

Adalimumab, the monoclonal anti-tumour necrosis factor antibody, has transformed outcomes in JIA-associated uveitis [59]. In the SYCAMORE trial, combination therapy of adalimumab with methotrexate halved treatment-failure rates compared with methotrexate alone, with treatment failure seen in 16 of the 60 (27%) of children in the treatment arm, versus 18 of the 30 children in the placebo (methotrexate alone) arm (60%) [21]. The ADJUVITE study corroborated these findings, demonstrating improved remission rates (9/14 children on adalimumab achieved control, or 64 versus 3/15 on placebo, 20%) with adalimumab initiation [19]. In a long-term observational cohort of 154 patients, clinical remission was achieved in 60.0% of the 95 adalimumab-treated patients versus 20.3% of the 59 on infliximab, further cementing adalimumab’s role as the first-line biologic therapy [60]. This study was however limited by the use of infliximab at a lower than typical dose, with children prescribed 5 mg/kg rather than the 6 -10 mg/kg used in other centres [61]. 

Although adalimumab is typically administered in set fortnightly doses (20 mg for those children weighing under 30 kg, and 40 mg for larger children and adults), adalimumab response may be further maximised through individualized dosing. Patients unresponsive to standard fortnightly administration may achieve remission when dosing increases to weekly intervals [62]. Anti-drug antibody assays may allow timely adjustments before clinical failure, and concurrent methotrexate may mitigate immunogenicity, preserving therapeutic effect [22, 63]. Despite the advances in care achieved with the use of anti-TNF treatment, long-term safety data are limited, and the low but significant infection risk is a concern. High drug costs and variable reimbursement policies may impede access and exacerbate disparities. The considerable variability in pharmacokinetics, immunogenicity and disease phenotype further highlights the importance of precision approaches.

Individualized approaches may also be necessary for successful treatment cessation. The ADJUST trial revealed the challenge of stopping adalimumab even after more than a year of quiescence, reporting disease relapses in 68% of patients (30/44 children) within 48 weeks, compared with 14% (6/34) who continued therapy [64]. For the 220 children within the UK JIA Biologics Register who managed to stop biologic therapy, preexisting JIA-U more than doubled the odds of relapse, with uveitis prevalences of 28% amongst the 118 who restarted biologic due to disease relapse, versus 4% amongst children who successfully remained off treatment (adjusted odds ratio 2.4 (95% confidence interval 1.2, 4.8) [65]. There is a need for proactive therapeutic-drug monitoring, standardised and validated tapering regimens, improved definitions of treatment remission and monitoring biomarkers to inform discontinuation plans. Future studies should aim to validate tapering protocols, predictive biomarkers and pharmacokinetic data to establish precision-medicine frameworks for anti-TNF use in JIA-U.

### New therapies: beyond anti-TNF agents

Tocilizumab, an interleukin 6 (IL-6) receptor antagonist, has a role to play in the management of adalimumab refractory JIA-U complicated by macular oedema, particularly when given by intravenous administration [66, 67]. Across a single arm study of subcutaneous tocilizumab, in which 21 children received either 162 mg every two or every three weeks (with body weights of more or less than 30 kg respectively), whilst only a third of children achieved disease control, 75% of the 4 children with macular oedema exhibited resolution of the oedema. In an earlier single arm study of IV tocilizumab, of the 17 children treated with 8 mg/kg every four weeks, disease control was achieved in 10 (59%) and all 5 cases of macular oedema had a successful response to treatment. Abatacept, a cytotoxic T-lymphocyte associated protein 4 (CTLA-4) analog, has also been recommended as a therapeutic option for children with disease refractory to anti-TNF [11]. However, studies suggest that the positive impact may be limited to a minority of refractive cases [17, 23, 66, 67]. Further studies will be needed to understand the optimal treatment regimens (dosing intervals and administration routes) for efficacy across different patient groups.

Research into alternative agents is underway. In refractory JIA-U, and non-JIA ANA positive anterior uveitis, Janus Kinase inhibitors (JAKi) have shown possible benefit, with significant reductions in inflammatory grade six months after commencement of baricitinib [68]. However, only a third of children (8 children of 24) with disease refractory to methotrexate and adalimumab achieved disease control with baricitinib within a recent multi-centre trial [68]. There was also poor response to baricitinib from children who were naïve to biologics, with only a fifth of those children (1/5 children) achieving disease control [68]. Improved identification of disease sub-types likely to response, or of predictors of therapeutic response will be needed for effective use of JAKi in JIA-U. Safety profiles were reasonable, with no evidence of cardiovascular adverse events, although these typically only occur in those at known cardiovascular risks or with long term use of JAKi agents [69]. Other JAKis, such as tofacitinib and ruxolitinib appear to have some effectiveness in JIA [70, 71], suggesting possible use in JIA-U.

There are hints of future therapeutic targets for JIA-U from basic science and biomarker studies of disease. Proteomic and transcriptomic profiling reveal prominent B-cell signatures in the AqH and iris tissue of affected patients [72], with elevated immunoglobulin transcripts, increased levels of B-cell survival factors, and higher concentrations of IL-6 compared to controls. There are a proportion of patients in whom ocular and serum sampling has revealed concordant elevations of S100A8/A9 and S100A12 in JIA-U [33]. This parallel rise of innate immune mediators supports the view that JIA-U involves both local ocular and systemic immune activity. Taken together, these findings highlight potential therapeutic targets involving B-cell, plasma cell and myeloid-driven inflammation. These studies also suggest the future use of serum and ocular fluid biomarkers in predicting therapeutic response, or in determining true disease remission.

There is also work underway to optimise an underused therapeutic approach in JIA-U: local disease therapy delivered into or around the eye. Local therapy has, up to now, been limited to corticosteroid delivery, which tend to only provide short term control, whilst conferring the risk of blindingly high eye pressures (glaucoma) and cataract. For example, dexamethasone intraocular implants (Ozurdex) induce quiescence in the majority of eyes within four weeks but carry an almost 20% risk of elevated intraocular pressure [73, 74]. More promising, for refractory cases complicated by macular oedema, is use of intravitreal fluocinolone acetonide implants such as Retisert, which demonstrated sustained remission for up to 18 months without serious adverse events, and transient IOP spikes with no resultant pressure related visual disability [75, 76]. Improved understanding of biomaterials and innovative delivery systems may also reawaken the interest in local treatment (intra, or periocular injections) with disease modifying agents such as methotrexate, or of biologic agents [77]. Delivery of such treatments for younger children, whilst avoiding the systemic risks of therapy, carries the burden of repeated general anaesthetic on global health and development outcomes [78]. 

### Evidence gaps in JIA-U: addressing the known unknowns on adult outcomes

Although JIA-U is diagnosed in childhood, it may persist as an active, sight-threatening condition well into adult life. Investigators have reported ongoing disease in between 24% and 56% of all adult patients more than a decade after onset, with ongoing activity seen in 19/78 young people 18 years after JIA diagnosis in one multicentre Nordic study [79, 80], and with activity noted in 28/52, 54% of 18 year olds in a Dutch study [8], and a recent meta-analysis of 1942 young people with JIA-U estimating the prevalence of active disease to be a minimum of 15% at 25 years and a maximum of 52% at 25 years [81]. This was despite systemic immunomodulation use in the majority. There was also frequent development of cataract and band keratopathy, cystoid macular oedema and glaucoma across these adult groups with childhood onset disease [9, 82]. The pattern of long term JIA-U outcomes may in future show a cohort effect following the relatively recent (i.e. since 2016) wider use of early intervention with biologic agents across children with JIA. In other words, we may see a lower incidence of blinding complications in early adulthood. The wider use of adalimumab in JIA may in some cases alter the natural history of JIA-U, with delayed rather than prevented onset. There may be (previously almost unheard of) new incident episodes of adult JIA-U activity in cases where biological agents are stopped following achievement of long term JIA disease control or remission.

While paediatric screening guidelines for JIA-U are well established, these cohorts expose the profound lack of adult-focused, longitudinal studies, and speak to the need for formalized transition-of-care pathways. Persistent active inflammation in adulthood, combined with intensive treatment burdens and high complication rates, demands cautious, multidisciplinary management.

### Evidence gaps in JIA-U: interventions for mental health burden

Juvenile idiopathic arthritis–associated uveitis imposes a profound, life-spanning burden on affected children as they transition into adulthood. Although Mendelian randomization analyses do not support direct genetic causality between uveitis and mood disorders, clinical observations document elevated rates of anxiety and depression amongst individuals with JIA-U [26]. Additionally, intriguing evidence of the association between systemic inflammation and mental health is emerging [83, 84], and future work should give insights into the multi-factorial picture of the determinants of mental health outcomes in this group. The challenge for clinicians now is to remain aware of the importance of identification of children at risk of poor psychosocial outcomes and to develop, validate and implement the early interventions needed to avoid the trajectory towards poor adolescent and adult mental health. The validation of the Effects of Youngsters’ Eyesight on Quality of Life (EYE-Q) questionnaire offers a uveitis-specific, vision-related quality-of-life instrument, facilitating routine psychosocial screening and timely referral in paediatric practice [85]. 

The burden of disease and disease care falls also on their families [26]. Empowering, co-developed resources, such as self-care passports with personalized action plans, and family focused disease education resources, have demonstrated enhanced self-management efficacy and disease understanding, illustrating the value of holistic, family-centred care frameworks [86, 87]. More work is needed to ensure that children affected by JIA-U, and their families, are supported across their disease odyssey.

## Conclusion

Juvenile idiopathic arthritis-associated uveitis (JIA-U) remains an important cause of visual impairment in children with autoimmune disease, marked by challenges across the lifecourse, from apparently silent onset in early childhood to long-term care. Disease often persists into adulthood, raising issues around continuity of care, treatment adherence, and the need for lifelong surveillance. Novel advances in JIA population stratification, using serological, genetic and ocular fluid biomarkers, will enable prompt detection of those at greatest risk of developing uveitis, ensuring that limited specialist ophthalmic services are directed towards those in greatest need. Novel imaging approaches will provide specialist level assessment to settings where such care is currently unavailable, enabling local delivery of care for families. Multimodal biomarker based patient stratification, encompassing genomics, proteomics and ‘radiomics’ (imaging based) may also help personalize therapy choices, and inform disease monitoring, as well as providing insight into disease mechanism and possible therapeutic targets. Adverse psychosocial outcomes, influence by factors such as the perceived and actual negative burden of invasive treatments, frequent absence from school, and risk of vision loss, remain under-addressed. New complex interventions such as co-developed educational materials, support tools and patient-reported outcomes tailored for pediatric uveitis may help, but more is needed.

## Data Availability

Data sharing is not applicable to this article as no datasets were generated or analysed during the current study.

## Abbreviations

AUC: Area under the receiver operated curve
AqH: Aqueous Humour
AS: Anterior segment
JAK: Janus Kinase
HLA: Human Leukocyte Antigen
IL-6: Interleukin 6
JIA-U: Juvenile Idiopathic Arthritis associated Uveitis
MRP: Myeloid reactive protein
OCT: Optical coherence tomography
OCTA: Optical coherence tomography angiography
TNF: Tumour necrosis factor
